# Supplementary material for: MANTA, an integrative database and analysis platform that relates microbiome and phenotypic data
Source: PLoS One. 2020 Dec 4;15(12):e0243609. doi: 10.1371/journal.pone.0243609 (PMC7717536; doi:10.1371/journal.pone.0243609)

**S1 Appendix. An illustration for importing data into MANTA basic.**

The user can upload the data from the 'Data management' function which can be accessed from the menu at upper right or if there is no data in the database, a link 'Click to upload the data' is shown in the empty table.


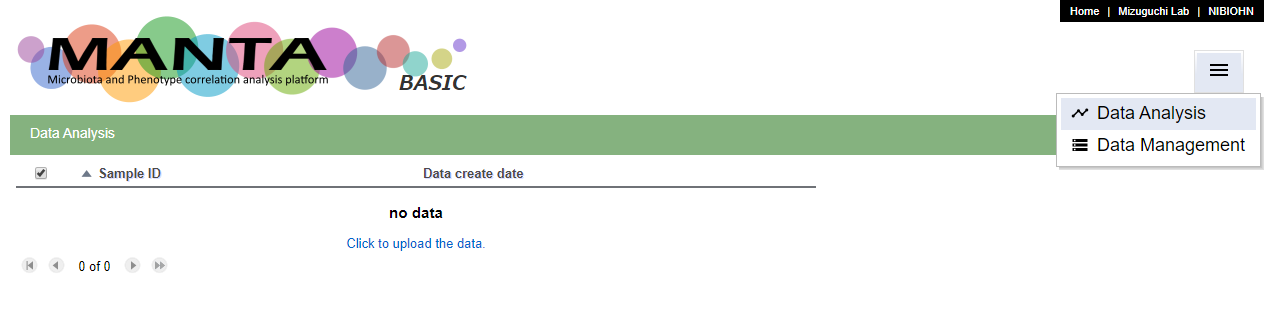


To upload the (phenotypic) parameter data, select 'Phenotype parameters' as the 'File type', and choose the data file to upload.


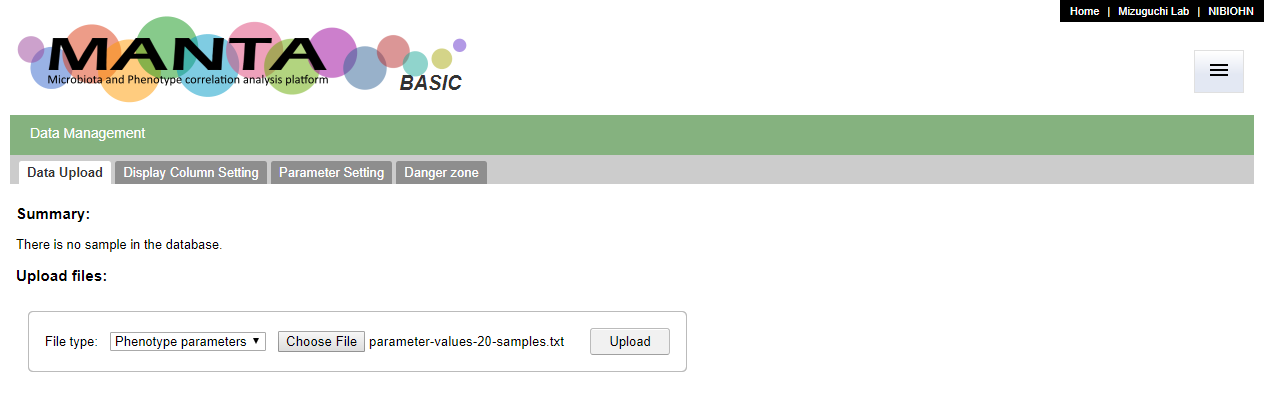


The file should be a tab delimited file. The first column should be the sample identifiers (ids) and the first row should be the header of different parameters. After clicking on the 'Upload' button, there is a dialog window showing the preview of the file. If the sample ids and the parameter header are allocated correctly, press 'OK' to finish data upload.


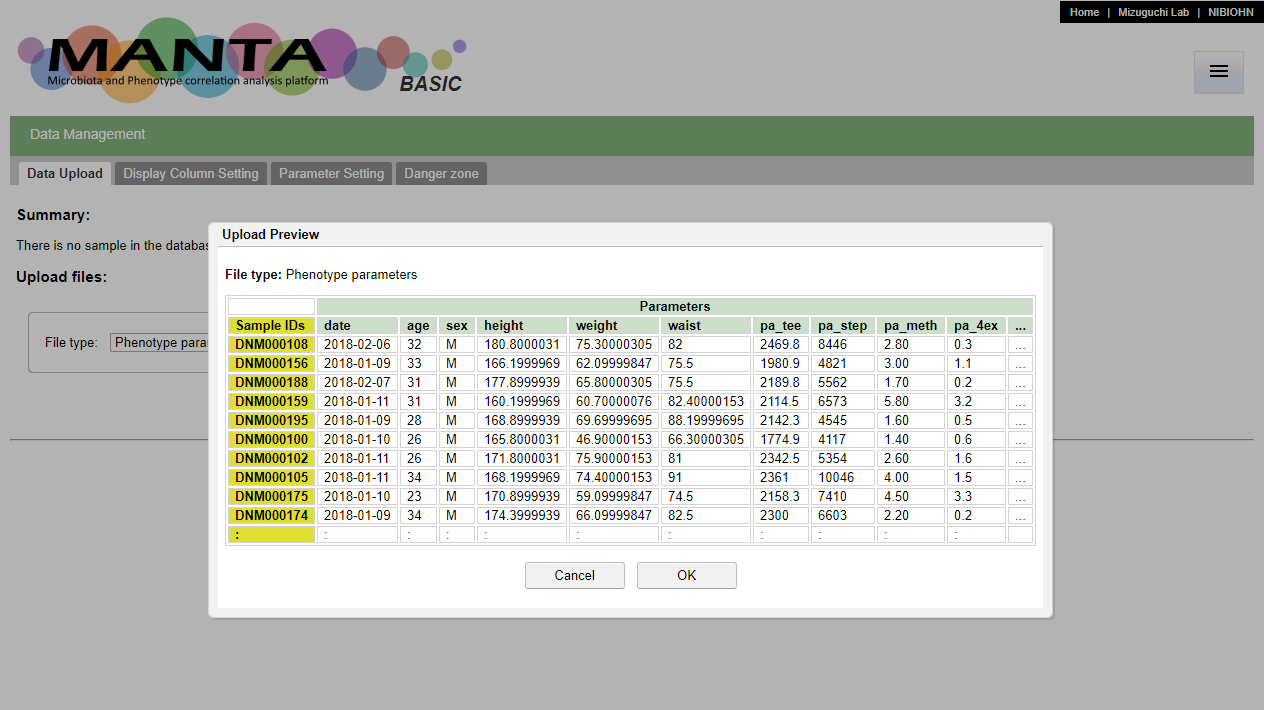


To upload the microbiome data, select 'Microbiota' as the 'File type', and choose the data file to upload.


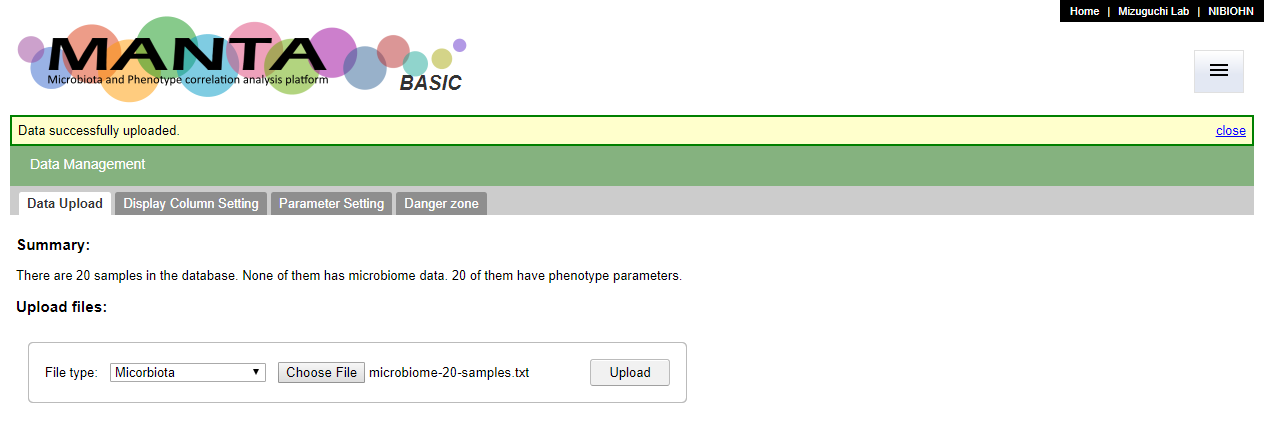


The file should be a tab delimited file. The first column should be the hierarchy of taxonomy from kingdom (e.g., Bacteria), separated by the semicolon. The first row should be the header of sample identifiers (ids).


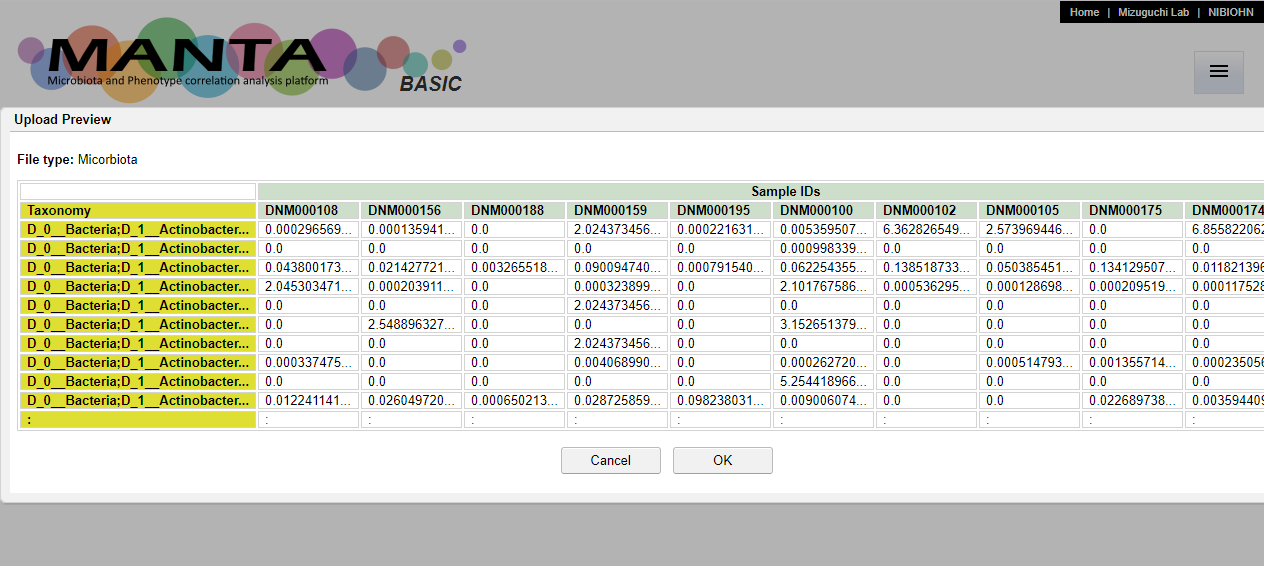


By default, all the uploaded parameters are set to the 'free text' type. The distance of the sample will be automatically calculated (Bray-Curtis and Jaccard index). The user can set the parameter types in the 'Parameter Setting' tab in the 'Data Management' function. Only the 'continuous variable' type could be used for calculating the correlation coefficient, and only the 'continuous variable', 'unranked category' and 'ranked category' could be used to color the data points in the PCoA plot.


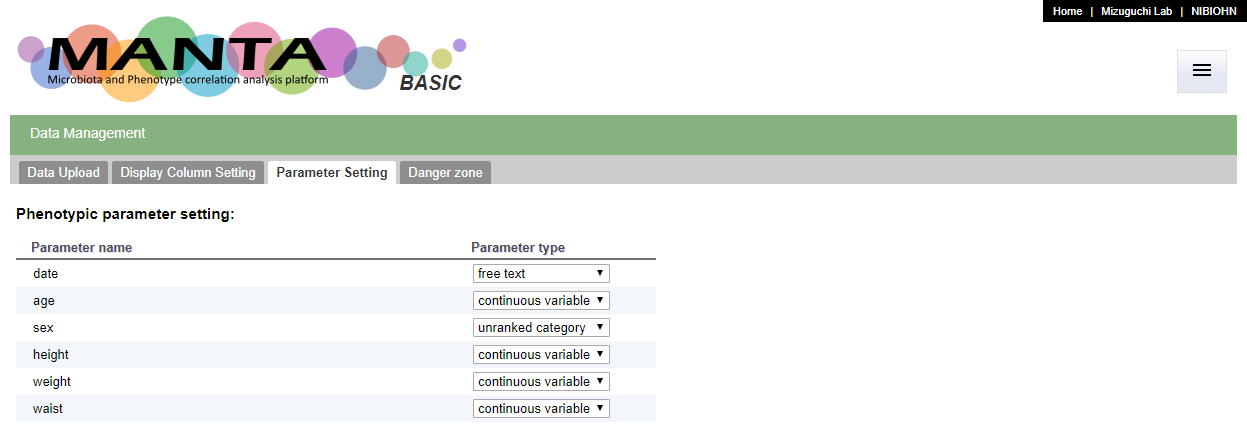

Supplement: S1 Appendix — (DOCX) [file pone.0243609.s001.docx]
