# Supplementary material for: MANTA, an integrative database and analysis platform that relates microbiome and phenotypic data
Source: PLoS One. 2020 Dec 4;15(12):e0243609. doi: 10.1371/journal.pone.0243609 (PMC7717536; doi:10.1371/journal.pone.0243609)
Supplement: Attachment — Submitted filename: manta-response-to-reviewers-2.docx [file pone.0243609.s003.docx]

**Response to Reviewers**

**Details:**

Reviewer #1: I appreciate the changes authors have made in the revised MS. Thank you for clarifying my queries and restructuring the manuscript. If possible, the authors must give a statement indicating their plans on how long is the tool expected to be supported and maintained given the funding they have. This is important because many tools after publication are forgotten by developers. I hope that this tool will be maintained and updated for at least 5-10 years.

Our current funding supports tool development and related activities for the next five years. We hope that we can continuously improve and maintain the MANTA project for at least 5-10 years.

Reviewer #2: This revision has addressed most of my concerns. I can use the online manta website and the offline software. I suggest improving the documentation by at least provide:
1) How to generate the microbiota file (what software, what file format is accepted)?
2) What file format is expected for user’s phenotype file?
3) Explain “Parameter type”. What does “others” mean? How does MANTA treat “others” compared to other types? The example phenotypic file has 290 variables, and I suggest automatically inferring the parameter types in MANTA.

Thank you for the suggestions. We have improved the online documentation to address those issues. The following explanation has been added:

1) The currently accepted microbiota file format is a tab-delimited table format (see the sample data as an example), which can be easily converted from the BIOM (The Biological Observation Matrix) format file (https://biom-format.org/). The BIOM format is adopted by several popular projects including QIIME and MG-RAST. Kraken/Bracken produces results in a different format and we are working on enabling the system to import this type of data.
2) At the moment, the accepted format is a tab delimited table format that contains different parameters in each column and different samples in each row.
3) The “others” type was designed for the data types that do not belong to any of the rest. At the moment, this type of data is treated in the same way as the type ‘free text’, and not used for any correlation calculation.
   Regarding the auto assignment of the data types, we will add this feature in a future release.

Reviewer #3: Upon this revision I now understand that the website described in the first version of the MANTA manuscript was totally fake--or I should say that it is a useless website only for demonstration purpose. The most critical point, as I identified, is that the installation procedure of the webpage components (jdk, tomcat, postgresql, data preprocessing, gwt compilation, to name just a few) can be much more challenging to users compared to ordinary pipelines. Due to this I do not know why users need to spend efforts to set up their own website--using command line pipelines can be easier than that. Thus, again, I doubt the very existence meaning of MANTA.

I have said it in my first review but allow me to say it again: please think and discuss with non-computational guys (or just let them try) in order to know their needs. Setting up a website can also be challenging even for people working on computational tasks. Please ponder the needs of non-computational people and design the system/pipeline accordingly.

Our demonstration database includes real-life (albeit small sample size) data. We believe that such a website, in line with many other open source projects, will be useful for showing key features of the software tool. We appreciate the hurdles for the non-computational users. In the revised version of the manuscript, we already discussed the importance of addressing the needs of non-technical users (in the paragraph starting from line 371), and this is exactly why we developed and released a standalone version named MANTA basic; the user can simply download a single all-in-one package and launch the application with a double click. To clarify these points, we have updated the documentation on our website.
